# Psychometric properties of the brief sense of community scale for urban-dwelling older adults

**DOI:** 10.1093/geront/gnaf239

**Published:** 2025-10-27

**Authors:** Max Bloem, Jane Murray Cramm, Anna Petra Nieboer

**Affiliations:** Department of Socio-Medial Sciences, Erasmus School of Health Policy & Management, Erasmus University Rotterdam, Rotterdam, the Netherlands; Department of Socio-Medial Sciences, Erasmus School of Health Policy & Management, Erasmus University Rotterdam, Rotterdam, the Netherlands; Department of Socio-Medial Sciences, Erasmus School of Health Policy & Management, Erasmus University Rotterdam, Rotterdam, the Netherlands

**Keywords:** Ageing-in-place, Sense of community, Urban communities, Instrument validation, Neighborhood

## Abstract

**Background and Objectives:**

As populations age and diversify, understanding older adults’ sense of community becomes critical for promoting their well-being. This study aimed to validate the Brief Sense of Community Scale (BSCS) among community-dwelling older adults of native Dutch and migrant backgrounds living in Rotterdam, the Netherlands.

**Research Design and Methods:**

A representative sample of 862 individuals aged 65 years and older completed the BSCS. The sample included 300 (34.8%) native Dutch, 211 (24.5%) Turkish-Dutch, 200 (23.2%) Surinamese-Dutch, and 151 (17.5%) Moroccan-Dutch participants. Psychometric properties were assessed through analyses of internal consistency, factorial validity, and measurement invariance across gender and ethnicity, based on established theoretical frameworks of sense of community.

**Results:**

Confirmatory factor analyses supported both the first-order and second-order four-factor models of the BSCS, with good model fit indices. For the second-order four-factor model, these were CFI = 0.97, RMSEA = 0.06, and SRMR = 0.027. The full scale showed strong internal consistency (Cronbach’s α = 0.88), with subscale reliabilities ranging from 0.64 to 0.88. Measurement invariance testing confirmed configural, metric, scalar, and (for gender) strict invariance, indicating that the BSCS functions equivalently across gender and the four largest ethnic groups in the Netherlands. These findings support the scale’s structural validity and cross-group comparability in diverse older adult populations.

**Discussion and Implications:**

The BSCS is a reliable and valid measure of sense of community for both native and migrant older adults. It offers valuable insights for research, policy, and initiatives aiming to improve social connectedness and well-being in multicultural urban settings.

Sense of community is a well-established concept in social science that describes how individuals perceive their belonging to, and meaningful membership in, collectives (Peterson et al., 2008; Sarason, 1974). McMillan and Chavis (1986) conceptualized sense of community as a multidimensional construct comprising four interrelated dimensions: needs fulfillment (the extent to which group membership meets personal needs), membership (a feeling of belonging or being part of a collective), influence (a sense of mattering within the group), and emotional connection (shared history, experiences, and emotional bonds). These dimensions continue to inform contemporary research on community and underpin the conceptual framework of established instruments such as the Brief Sense of Community Scale (BSCS).

Several tools have been developed to measure this construct. The original Sense of Community Index (SCI), developed by Chavis et al. (1986), has been widely used in both academic and applied settings. However, the SCI has been critiqued for limitations in psychometric robustness, leading to the development of the SCI-2, which expands upon the original dimensions with more items to enhance reliability (Chavis et al., 2008; Long & Perkins, 2003; Obst & White, 2004; Peterson et al., 2006). While these instruments offer comprehensive coverage, their length and complexity can be barriers to use, especially in older populations or multicultural survey contexts.

The BSCS, developed by Peterson et al. (2008), provides a concise and theoretically grounded alternative. It consists of only eight items, two per dimension, around McMillan and Chavis’s framework. Its brevity makes it particularly well-suited for large-scale surveys and use among older adults, including those with lower literacy levels or cognitive burdens. It has also demonstrated strong factorial validity across a range of populations and in neighborhood research specifically, though evidence among ethnically diverse older adults remains limited (Buckley et al., 2022; Chiessi et al., 2010; Griffin et al., 2022; Lardier et al., 2018). This study aims to address this gap by validating the BSCS among older adults of both native Dutch and migrant backgrounds in an urban European context.

Each of the four dimensions of the BSCS is based on ­McMillan and Chavis’s (1986) framework and is theoretically linked to key mechanisms by which community life contributes to individual well-being. The dimension of needs fulfillment captures the degree to which individuals perceive that their personal needs are met through their connection to the community. This aligns with Lindenberg’s (1996) theory of well-being, which views social environments as important sources for meeting basic needs such as comfort, stimulation, and behavioral confirmation. The membership dimension refers to the feeling of belonging and being part of a clearly defined group, which is central to theories of social identity and emotional safety. The influence dimension reflects the sense that one can contribute to and be affected by the group. It emphasizes mutual responsibility and is closely connected to perceptions of agency and participation. This is particularly relevant in older populations who may face barriers to influence their local context. Finally, emotional connection concerns the presence of shared history, common experiences, and emotional bonds among members of a community. Taken together, the four dimensions provide a comprehensive theoretical basis for assessing how community ties function in the everyday lives of older adults.

As societies face demographic shifts, including population aging, urbanization, and increasing (ethnic) diversity, an understanding of the sense of community has become particularly relevant. Although these broader changes occur at national and global scales, their effects are often felt most acutely at the neighborhood level, where individuals live and interact daily. Neighborhoods are widely recognized as significant determinants of health and well-being (Ige-Elegbede et al., 2022), especially for older adults (Padeiro et al., 2022), and are important sources of a sense of community (Glynn, 1986).

Cities, especially those in many Western countries, are becoming increasingly ethnically diverse (Phillimore et al., 2020; Vertovec, 2007, 2023). This increased urban diversity is likely to influence native and migrant inhabitants’ sense of community (Townley et al., 2011). For example, many older migrants have transnational ties, and they may prefer to maintain relationships within ethnic enclaves, leading to differences in their neighborhood sense of community experiences from those of native-born individuals. The neighborhood sense of community of native individuals may be stronger due to their nativity and identification with the broader national culture but may be negatively impacted by ethnic diversity and outgroup size (Castellini et al., 2011; Mannarini et al., 2017; ­Stewart & Townley, 2020; Townley et al., 2011).

An understanding of the sense of community in neighborhoods is important to support the creation and maintenance of age-friendly communities. Drawing on Lindenberg’s (1996) theories of well-being and community, neighborhood communities can be conceptualized as localized networks where individuals collectively fulfill multiple well-being needs, including comfort, stimulation, affection, behavioral confirmation, and status (Nieboer et al., 2005; Völker et al., 2007). In age-friendly communities, the sense of community functions as a mechanism through which older adults achieve physical and social well-being collectively in their neighborhoods. To evaluate the capacity of neighborhoods to promote well-being and address the varied needs of their older residents, a reliable and culturally sensitive measure of the sense of community is required.

The significance of the sense of community for older adults extends beyond their subjective well-being (Stewart & ­Townley, 2020). Empirical research has shown that a strong sense of community is linked to better mental and physical health (Michalski et al., 2020), enhanced social involvement (Tang et al., 2017), and greater resilience (Zhang et al., 2018). The sense of community can safeguard urban-dwelling older persons, particularly those with migrant backgrounds, from the obstacles of aging in potentially isolated situations. In contrast, a lack of this sense may intensify feelings of alienation and loneliness, which are common in this group (Fokkema & Ciobanu, 2021; Stephens & Phillips, 2022). The further development of research on these themes requires a robustly validated measure of the sense of community for this population.

This study addresses the underexplored validity of a sense of community measure (the Brief Sense of Community Scale) among older adults, particularly those with migration backgrounds. The reliability and validity of the measure were explored across four urban-dwelling groups in the Netherlands (native-Dutch, Surinamese-Dutch, Turkish-Dutch, and Moroccan-Dutch) to capture the experiences of these populations and enable comparison. This research highlights the importance of the use of culturally sensitive tools to understand the sense of community and its role in promoting well-being among older adults in diverse urban settings. The study aimed to assess whether the BSCS is a reliable and valid instrument for measuring sense of community in this population and to test whether the scale demonstrates measurement invariance across gender and ethnic groups. We hypothesized that the BSCS would show good internal consistency, confirmatory factor structure, and invariance across these groups, supporting its use in multicultural aging research.

## Method

### Procedure and participants

The data for this work were collected as part of a broader study examining how older adults foster well-being in their neighborhoods in Dutch cities (Nieboer & Cramm, 2022). Before distribution, the survey used in the study was rigorously pre-tested to ensure its validity and reliability. In pilot testing, the survey was administered to a small group of participants to identify potential issues, and cognitive interviews were conducted to better understand how participants interpreted and responded to the questions. Additionally, native speakers of the languages of the target populations, including Turkish and Arab, carefully translated the survey to ensure cultural appropriateness and clarity.

Older adults (aged ≥65 years) from Native-Dutch, Turkish-Dutch, Surinamese-Dutch, and Moroccan-Dutch backgrounds were recruited using a stratified proportional sampling strategy with quotas. The sampling frame consisted of 72 neighborhoods within 10 districts of Rotterdam. Four strata were defined by ethnic background, based on the individual’s own country of birth. Within each ethnic stratum, participants were proportionally selected from neighborhoods according to the share of that group among older adults (65+) in each neighborhood, as indicated in municipal demographic data. For example, the 500 Surinamese participants were drawn proportionally from neighborhoods such that the number selected from each neighborhood reflected the relative concentration of Surinamese older adults in that area compared with the citywide total. The same approach was used for the other three groups. Quotas of approximately 500 individuals per ethnic group ensured adequate subgroup sizes for analysis. This procedure enhanced representativeness while ensuring replicability of the study design.

The initial sample of potentially eligible participants (*n* = 1998) was drawn from the municipal register of Rotterdam. These individuals first received an introductory letter explaining the survey’s purpose and significance. A follow-up reminder letter was sent 1 month later to encourage participation. Subsequently, individuals were contacted by telephone to further engage them, and field workers visited them to assist with survey completion when needed.

Monetary incentives of €5, €10, or €15 were randomly assigned to participants to test different incentive strategies in this pilot study context. Initial analyses showed only minor differences in response patterns across incentive amounts. Given the random assignment, we do not expect the variation in incentives to have introduced significant bias in the results. Data were collected between February and August 2023, with respondents completing the survey via paper, online, or with field workers’ assistance at home.

## Measures

### Sense of community

Participants’ sense of community was measured using the Brief Sense of Community Scale (BSCS). The concept of the sense of community was originally introduced by Sarason (1974) and systematically developed and empirically tested by McMillan and Chavis (1986). The resulting multidimensional concept encompasses four dimensions: (a) needs fulfillment, (b) group membership, (c) influence, and (d) emotional connection. Historically, (urban) neighborhoods have been identified as sources and locations of the sense of community (Glynn, 1986). The BSCS has eight items referring to respondents’ neighborhoods, loading on the four subscales (see Supplementary Material). Responses are structured by a 5-point Likert scale ranging from “completely disagree” to “completely agree.”

The factor structure and underlying theory of the BSCS have been confirmed in the context of neighborhood communities (Peterson et al., 2008). The instrument has been used in many different contexts and populations, including with military service members (Wombacher et al., 2010), adolescents (Chiessi et al., 2010), and the LGBT community (Griffin et al., 2022). The BSCS has also undergone validation in various international settings and languages (Lardier et al., 2018; Sočan & Kobal Grum, 2023; Yu et al., 2022). Its robust theoretical underpinning, widespread adoption, and conciseness make it suitable for use in the study of older adults’ sense of community.

The validation of the BSCS for use with older adults has been limited. Buckley et al. (2022) validated the instrument for use with Spanish-speaking older adults in Puerto Rico, but that work had two shortcomings. First, the authors used a nonprobability sampling strategy, which restricts the generalizability of their results, may have introduced selection bias, and reduces representativeness. Second, their sample of 154 participants is considered to be modest for instrument validation; it may have led to errors in the estimation of psychometric features such as internal consistency and factor structure. Samples of 200–500 participants are recommended for instrument development and validation to guarantee acceptable statistical power (e.g., Costello & Osborne, 2005).

### Well-being

Well-being was measured using the 15-item Social Production Function Instrument for the Level of Well-Being (SPF-ILs) (­Nieboer et al., 2005). This theory-driven instrument is based on a model of well-being needs derived from Lindenberg’s (1996) social production function theory, whereby overall well-being is produced by the meeting of physical and social well-being needs. Physical well-being is produced by the realization of the instrumental goals of comfort and stimulation, and social well-being is produced by the realization of the instrumental goals of status, behavioral confirmation, and affection. SPF-ILs scores range from 1 (*never*) to 4 (*always*). This instrument has been validated extensively, including for native-Dutch older adults and older adults with migration backgrounds (Nieboer & Cramm, 2018).

### Self-rated health

Respondents rated their health (“In general, how is your health?”) on a scale ranging from 1 (*excellent*) to 5 (*poor*). The variable was recoded so that higher scores reflected better self-rated health.

### Loneliness

Loneliness was measured with the question “How often do you feel lonely?” Responses ranged from 1 (*never*) to 3 (*often*).

## Analyses

### Confirmatory factor analysis

**Figure 1.**
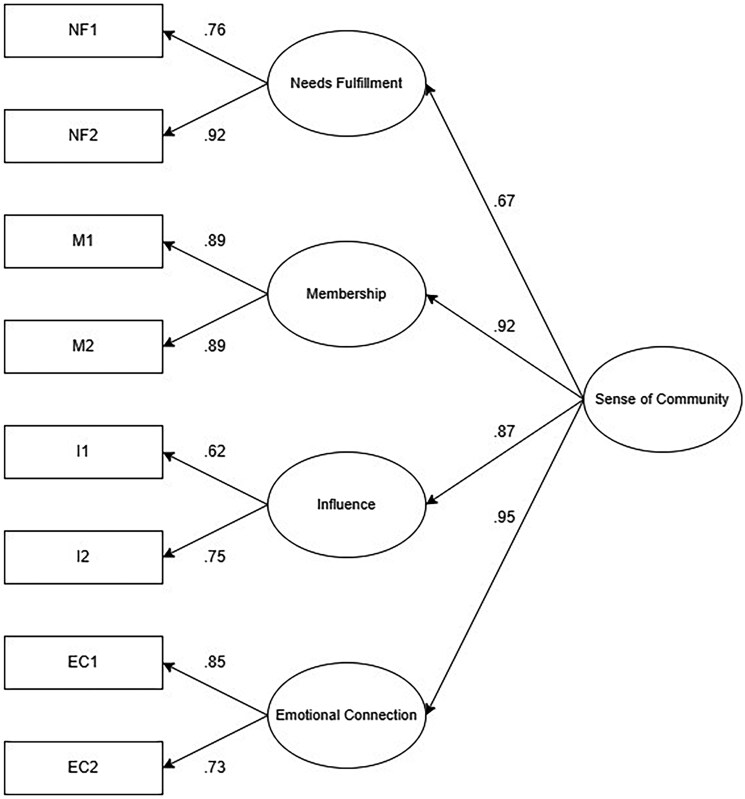
Confirmatory factor analysis results with standardized factor loadings for the second-order four-factor model. *Note*. NF = Needs Fulfillment; M = Membership; I = Influence; EC = Emotional Connection.

Confirmatory factor analysis (CFA) was performed using the lavaan package (Rosseel, 2012) in R (R Core Team, 2021). A single-factor model (model 1), a four-factor model (model 2), and a second-order four-factor model (model 3) were assessed, in line with previous validation studies (Buckley et al., 2022; Peterson et al., 2008). The second-order model posits that the four subscales load onto a higher-order latent factor representing the overall sense of community (see Figure 1). To fit the models, robust maximum likelihood (MLR) and full information likelihood (FIML) estimation were used. MLR estimation is more reliable in cases of multivariate normality violations (Satorra & Bentler, 1994). FIML estimation utilizes all available data, avoiding listwise deletion and thereby providing more accurate parameter estimates in cases of missing data (Enders & Bandalos, 2001). Model fit was assessed using the Satorra–Bentler *χ*^2^ test, root mean square error of approximation (RMSEA), comparative fit index (CFI), and standardized root mean square residual (SRMR) with standard cut-off values (good, RMSEA < 0.06, CFI > 0.95, and SRMR ≤ 0.08; acceptable, RMSEA < 0.08 and CFI > 0.90) (Byrne, 2016; Hu & Bentler, 1999; MacCallum et al., 1996).

### Reliability assessment

The internal consistency of the overall BSCS was assessed using Cronbach’s *α*, with 0.70 serving as the threshold for acceptable fit (Nunnally & Bernstein, 1994). In line with previous validation studies, we also examined the reliability of the instrument’s four two-item subscales (Buckley et al., 2022; Lardier et al., 2018; Peterson et al., 2008).

### Construct validity assessment

The validity of the BSCS was assessed by computing Pearson correlations of BSCS scores with self-rated health, well-being, and loneliness scores (see Table 5). The scale’s convergent ­validity was assessed with variables reflecting self-rated health and well-being (SPF-ILs). Research has shown that the sense of community is related positively to health and well-being, including among older adults (Michalski et al., 2020; Stewart & Townley, 2020). The sense of community is likely related more strongly to well-being than to self-rated health, as the SPF-ILs captures social aspects of well-being explicitly (Nieboer et al., 2005). In addition, the sense of community has been shown to be related more strongly to mental well-being then to general health, which the well-being instrument may capture more closely (Michalski et al., 2020). We also assessed convergent validity using loneliness, given its well-documented negative association with a sense of community in various populations, including older adults (Glass, 2020; Pretty et al., 1994; Prezza et al., 2001).

### Measurement invariance evaluation

We tested measurement invariance by gender and ethnicity to confirm model consistency across groups. This step was essential to confirm that the instrument measured the intended constructs consistently, regardless of group membership. Gender invariance was measured because aging processes and their implications for well-being have been shown to differ between older men and women, especially when considering social connection and interaction (Kiely et al., 2021; Nakash et al., 2022). Invariance according to respondents’ migration backgrounds was measured because research has revealed substantial differences in loneliness, happiness, physical health, and neighborhood conditions between native and immigrant older adults (Fokkema & Naderi, 2013; Greft et al., 2016; Jang et al., 2023; Jiang & Renema, 2021).

Configural, metric, scalar, and strict invariance was assessed. Configural invariance was evaluated by examining factor structure similarity; metric invariance was evaluated by examining the consistency of factor loadings; scalar invariance was evaluated by examining the extent to which item score differences reflected true latent differences, rather than biases; and strict invariance was evaluated by examining the similarity of measurement errors across groups.

## Results

The response sample (*n* = 862) consisted of 300 (34.6%) native-Dutch, 211 (24.5%) Turkish-Dutch, 200 (23.2%) Surinamese-Dutch, and 151 (17.5%) Moroccan-Dutch older adults. Fifty-six percent of the respondents were women. The respondents were aged 65–98 years [mean (*M*) = 73.4, standard deviation (*SD*) = 6.62] years and had 0–25 (*M* = 9.3, *SD* = 6.0) years of education. The total sample consisted of 862 participants, of whom 56% were women. The average age was 73.4 years (*SD* = 6.6). Gender and age distributions were generally comparable across ethnic subgroups. Among Native-Dutch respondents (*n* = 300), 56% were female and the mean age was 74.6 years (*SD* = 7.4). Among Surinamese-Dutch (*n* = 200), 61% were female with a mean age of 72.0 years (*SD* = 5.9); among Turkish-Dutch (*n* = 211), 53% were female with a mean age of 72.6 years (*SD* = 5.9); and among Moroccan-Dutch participants (*n* = 151), 54% were female with a mean age of 74.0 years (*SD* = 6.1). In total, 236 individuals were excluded from the sample due to relocation (*n* = 27), incorrect addresses (*n* = 24), difficulty speaking (*n* = 18), severe illnesses (*n* = 73), residence abroad or in institutions (*n* = 81), or death (*n* = 13). Ultimately, 862 participants completed the survey, yielding a final response rate of 49%. The response rate of 49% was calculated based on the remaining contactable case.

Participant and BSCS item characteristics are provided in Tables 1 and 2, respectively. Percentages of missing data ranged from 3.6% to 5.5%. Mean BSCS item scores ranged from 2.69 to 3.62.

**Table 1.** Sample characteristics.

Characteristics	Total sample (*n* = 862)
**Sex (female)**	56%
**Age (years)**	73.4 (6.6)
**Education (years)**	9.3 (6.0)
**Marital status (married)**	31%
**Household size**	1.6 (1.0)
**Ethnic background**	
***Native Dutch***	35% (*n* = 300)
***Surinamese***	23% (*n* = 200)
***Turkish***	24% (*n* = 211)
***Moroccan***	19% (*n* = 151)

**Table 2.** Brief Sense of Community Scale item characteristics.

Subscale	Statement	Valid responses (*n*)	Missing data (%)	Mean score (*SD*)
**Needs fulfillment**	1. I can get what I need in this neighborhood.	831	31 (3.6)	3.62 (1.02)
	2. This neighborhood helps me fulfill my needs.	827	35 (4.1)	3.36 (1.03)
**Membership**	3. I feel like a member of this neighborhood.	825	37 (4.3)	3.52 (0.96)
	4. I belong in this neighborhood.	823	39 (4.5)	3.59 (0.93)
**Influence**	5. I have a say about what goes on in my neighborhood.	815	47 (5.5)	2.69 (1.01)
	6. People in this neighborhood are good at influencing each other.	818	44 (5.1)	3.21 (0.91)
**Emotional connection**	7. I feel connected to this neighborhood.	827	35 (4.1)	3.57 (0.99)
	8. I have a good bond with others in this neighborhood.	828	34 (3.9)	3.58 (0.73)

### Measurement model findings

Measurement model findings are shown in Table 3. The single-factor model demonstrated poor fit across all indicators, failing to meet the thresholds for acceptable or good model fit. The four-factor model showed good model fit according to all indicators except the Satorra–Bentler χ^2^ value, which was significant, likely due to the sample size. The second-order model showed acceptable fit according to the RSMEA and good fit according to the CFI and SRMR.

**Table 3.** Model fit indices for the BSCS.

	Models		
Measures of fit	1 factor	4 factors	Second-order
***SB* (*χ*²)**	281.612	39.560	64.090
***df***	20	14	16
***P value***	0.000	0.000	0.000
***RMSEA***	0.125	0.047	0.060
***90% CI RMSEA***	0.115–0.134	0.034–0.052	0.048–0.072
***CFI***	0.855	0.986	0.973
***SRMR***	0.065	0.018	0.027

*Note.* SB *χ*2 = Satorra–Bentler chi-squared value, df = degrees of freedom, RMSEA = root mean square error of approximation, CI = confidence interval, CFI = comparative fit index, SRMR = standardized root mean square residual.

To determine the best-fitting model, we considered both statistical fit and the theoretical aim of the study, which was to measure the overall sense of community. Although the four-factor model fit the data well, the second-order model was preferable as it captures the hierarchical structure of the sense of community, reflecting both the four subdimensions and the overarching latent construct. Combined with the acceptable to good fit of the indices, this justified our choice to use the second-order model for all subsequent analyses. Accordingly, the following reliability, validity, and measurement invariance results are based on the second-order model.

Figure 1 shows the full factor structure and standardized factor loadings for the second-order four-factor model. Factor loadings from the individual items to the first-order latent variables ranged from 0.62 to 0.92 and were significant (*p* < .001). Factor loadings from the first-order latent variables to the second-order variable (sense of community) ranged from .67 to .95 and were significant (*p* < .001).

### Reliability

Reliability scores (Cronbach’s *α* values) for the instrument and its subscales are provided in Table 4. The BSCS showed good internal consistency reliability (*α*  =  0.88), as did the subscales of needs fulfillment (*α*  =  0.82), membership (*α*  =  0.88), and emotional connection (*α*  =  0.76). The influence subscale showed near-acceptable reliability (*α*  =  0.64).

**Table 4.** Reliability scores for the Brief Sense of Community Scale.

Factor	Cronbach’s *α*
**Needs fulfillment**	0.82
**Membership**	0.88
**Influence**	0.64
**Emotional connection**	0.76
**Overall**	0.88

**Table 5.** Pearson’s coefficients of correlation with Brief Sense of Community Scale total and subscale scores.

Variables^a^	Sense of Community	Needs fulfillment subscale	Membership subscale	Influence subscale	Emotional connection subscale	Well-being (SPF-ILs)	Loneliness	Self-rated health
**Sense of Community**		0.75***	0.98***	0.96***	0.99***	0.32***	−0.22***	0.12***
**Needs fulfillment subscale**			0.71***	0.70***	0.70***	0.29***	−0.18***	0.14***
**Membership subscale**				0.90***	0.95***	0.29***	−0.21***	0.09**
**Influence subscale**					0.94***	0.33***	−0.23***	0.14***
**Emotional connection subscale**						0.32***	−0.21***	0.11***
**Well-being (SPF-ILs)**							−0.45***	0.52***
**Loneliness**								0.38***
**Self-rated health**								

a Note. ***p < .001.

### Construct validity

As expected, BSCS scores correlated positively with well-being (*r* = 0.32) and self-rated health (*r* = 0.12) scores and negatively with loneliness (*r* = −0.22) scores (all *p* < .001).

### Measurement invariance

BSCS scores showed strict measurement invariance across genders, indicating that the measurement model has the same factor structure, factor loadings, intercepts, and residual variance for men and women (Table 6). They showed scalar measurement invariance across migration backgrounds, indicating that the measurement model has the same factor structure, factor loadings, and intercepts for all groups. However, residual variance may vary among these groups.

**Table 6.** Measurement invariance of the Brief Sense of Community Scale.

Model	SB χ²	df	*p*-Value	RMSEA	90% CI RMSEA	CFI	SRMR
**Gender**							
***Configural***	95.259	39	0.000	0.059	0.047–0.071	0.970	0.035
***Metric***	108.239	42	0.000	0.061	0.050–0.073	0.966	0.038
***Scalar***	115.087	47	0.000	0.059	0.047–0.070	0.965	0.039
***Strict***	135.667	55	0.000	0.059	0.049–0.069	0.958	0.042
**Ethnic background**							
***Configural***	184.240	85	0.000	0.075	0.062–0.087	0.951	0.067
***Metric***	193.968	94	0.000	0.071	0.059–0.083	0.950	0.068
***Scalar***	223.705	109	0.000	0.071	0.059–0.082	0.943	0.076
***Strict***	261.573	133	0.000	0.068	0.058–0.078	0.936	0.081

*Note.* SB χ^2^ = Satorra-Bentler chi-squared value, df = degrees of freedom, RMSEA = root mean square error of approximation, CI = confidence interval, CFI = comparative fit index, SRMR = standardized root mean square residual.

## Discussion

The results of this study confirm that the BSCS is a valid and reliable tool for the measurement of the sense of community of urban-dwelling older adults with and without migration backgrounds. The CFA demonstrated the good fit of the first- and second-order four-factor models, in line with original theory concerning the sense of community, the initial testing of the BSCS, and its subsequent validation for different populations (Buckley et al., 2022; Chiessi et al., 2010; Griffin et al., 2022; McMillan & Chavis, 1986; Peterson et al., 2008). Depending on the specifics of the research, either of these models can be used to measure the sense of community.

Our analysis confirmed the instrument’s internal consistency. The full BSCS exhibited good reliability, indicating that it is a reliable measure of the sense of community in the study population and suggesting that it suitably captures this multidimensional construct among older adults in general. The needs fulfillment and membership subscales also demonstrated strong reliability, aligning with research highlighting these dimensions as core components of the sense of community (Peterson et al., 2008). The strong reliability of the full scale and the needs fulfillment and membership subscales fits well with previous research that shows how important social connections are for meeting basic needs and feeling part of a group (Berkman et al., 2000). These social ties help older adults get support, stay engaged, and access resources, which all contribute to better health and well-being (Antonucci et al., 2014).

The emotional connection subscale showed acceptable reliability, which may point to variability in individuals’ experiences of emotional ties in their communities. This variability is understandable given that emotional bonds are deeply personal and influenced by numerous factors such as personality, cultural background, and life experiences. Earlier research emphasizes that emotional attachment can fluctuate based on how meaningful and reciprocal these ties feel (Baumeister & Leary, 1995). Moreover, aging itself can alter emotional priorities and social goals. Socioemotional selectivity theory (Carstensen, 1992, 2021) suggests that older adults often focus more on emotionally meaningful relationships, which might vary widely between individuals depending on their social circumstances. Moreover, the degree to which older adults source these meaningful relationships within their neighborhood, compared to other sources such as family, may differ substantially. Thus, the diversity in emotional connection scores likely reflects real differences in how older adults relate to and feel supported by their neighborhood communities, influenced by both personal and environmental contexts. Socioemotional selectivity theory (Carstensen, 1992, 2021) posits that older adults prioritize emotionally meaningful ties as they age. Our observation of variability in the *Emotional Connection* subscale scores fits this theoretical perspective, as older adults may differ in whether they seek these ties primarily from neighbors or from family and other networks. Thus, the diversity in scores reflects differences in how socioemotional goals are pursued in everyday community life.

The influence subscale exhibited less reliability, with a Cronbach’s *α* value falling slightly below the commonly accepted threshold of 0.70. This result suggests that the items of this subscale do not capture the construct of influence as consistently as the other dimensions are captured, and such underperformance was also observed in another validation study conducted with older adults (Buckley et al., 2022). The mean score for item 5 (“I have a say about what goes on in my neighborhood”) was substantially lower than were the other item scores, possibly because perceived community influence is more context dependent or variable among older adults. Research indicates that older adults’ sense of influence or agency within their neighborhoods can fluctuate due to factors such as health status, mobility, and social roles, making it a complex and dynamic construct (Buffel et al., 2012, 2013; Glass & Balfour, 2003; Hand et al., 2020). This variability could explain why the influence subscale items do not perform as reliably as other dimensions, reflecting diverse experiences among older adults.

Given the limited and context-specific nature of perceived influence, more research is needed to explore how older adults exercise agency in their communities, and how neighborhood environments support or hinder this sense of control. Increasingly researchers highlight this gap, calling for studies that theorize older adults as active agents in their own neighborhoods, and not just subjects affected by their environment (­Stephens & Allen, 2022; Wahl et al., 2012).

The construct validity analysis provided further support for the instrument’s reliability for the assessment of the sense of community. As expected, BSCS scores demonstrated significant positive correlations with well-being and self-rated health scores. These findings align with theoretical expectations and previous research suggesting that a stronger sense of community is associated with better psychological and physical health (Cramm & Nieboer, 2013; Gattino et al., 2013; Michalski et al., 2020; Stephens & Phillips, 2022; Stewart & Townley, 2020). This connection is grounded in ecological and social capital theories, which posit that individuals embedded in supportive social environments benefit from resources such as emotional support, shared norms, and collective efficacy, all of which promote health and resilience (Ayalon & Levkovich, 2019; Kawachi & Berkman, 2001; Norstrand & Xu, 2012; Putnam, 2020). Moreover, sense of community may buffer stress by providing coping mechanisms through social ties, enhancing both mental and physical health (Antonucci et al., 2014; Cohen & Wills, 1985). The observed correlations with well-being, health, and loneliness can be understood in light of ecological models of aging (Wahl et al., 2012), which emphasize that individual outcomes are shaped by the interaction between people and their environments. A stronger sense of community may provide both social resources and environmental supports that protect against loneliness and foster well-being. Likewise, social capital theory (Kawachi & Berkman, 2001; Putnam, 2020) helps explain why BSCS scores were linked to better outcomes: supportive networks, shared norms, and collective efficacy constitute forms of capital that enhance resilience and health.

The observed difference in health and well-being effect sizes was also anticipated. Earlier studies showed that the sense of community contributes more to mental health (Michalski et al., 2020), which the well-being instrument may better capture, than to general health (Kawachi & Berkman, 2001). Physical health may be influenced by a wider range of factors beyond social environment, such as genetics and lifestyle, which could minimize the direct impact of sense of community.

Conversely, BSCS scores correlated negatively with loneliness scores, reinforcing the notion that a robust sense of community mitigates loneliness by fostering meaningful interpersonal relationships and reducing social isolation (Glass, 2020; Pretty et al., 1994). This link is particularly critical given that loneliness has emerged as a major public health concern, especially among older adults, with well-documented associations with morbidity, mortality, and cognitive decline (Courtin & Knapp, 2017).

Taken together, these findings provide strong evidence for the construct validity of the BSCS and highlight the broader importance of social context in shaping health outcomes among older adults. They also suggest that efforts to foster a sense of community could be a valuable public health strategy to enhance well-being and reduce loneliness, particularly in aging populations.

The results of the measurement invariance analysis provide strong support for the robustness and comparability of BSCS scores across different demographic groups. The finding of strict measurement invariance across genders indicates that the measurement model functions equivalently for men and women. Specifically, it suggests that the factor structure, factor loadings, intercepts, and residual variance are consistent for both genders, ensuring that the instrument is equally valid and reliable for male and female respondents. This is a particularly meaningful result given the well-established gender differences in social roles, networks, and experiences of community across the life course (Fiori et al., 2006; Kiely et al., 2021; Moen & Chermack, 2005; Nakash et al., 2022).

Women tend to maintain larger and more emotionally close social networks than men, including in later life, which can influence how they engage with and perceive their communities (Blieszner et al., 2019; McLaughlin et al., 2010; Zhaoyang et al., 2018). These differences could, in theory, shape how individuals interpret or experience dimensions of the sense of community, such as emotional connection or influence. That the BSCS operates equivalently across genders suggests that it captures the construct of community in a way that is not biased by these differing relational patterns. It also supports the cross-gender applicability of the BSCS in future research and policy efforts aimed at understanding and enhancing community belonging in later life.

Similarly, the BSCS showed scalar measurement invariance across respondents with different ethnic backgrounds, suggesting that its factor structure, factor loadings, and intercepts are equivalent for these groups. This finding indicates that the instrument measures the same underlying constructs in the same way for groups with diverse ethnic backgrounds. However, the observation that residual variance may differ among ethnic groups warrants attention. It may indicate that certain aspects of the instrument’s measurement are more variable or sensitive to individual differences within specific ethnic groups. Differences in ageing processes and neighborhood perceptions between ethnic groups have been demonstrated in earlier studies (Cheng & Nicklett, 2022; Law et al., 2025). Moreover, it is likely that systematic forms of (neighborhood) exclusion, faced disproportionately by older migrants, impacts the perceptions of community (Ferraro & Shippee, 2009; Gee et al., 2019). Future research might explore the sources of this variation to better understand the specific factors that might influence the responses of members of these groups.

The findings of this study further emphasize the important link between older adults’ sense of community in their neighborhoods and their well-being. In theoretical terms, the sense of community concept used in this study aligns closely with the social production function theory, which suggests that individuals strive to fulfill their physical and social well-being needs through goal-directed behaviors (Lindenberg, 1996; Nieboer et al., 2005). The Needs Fulfillment subscale reflects how neighborhoods contribute to overall subjective well-being. The Membership and Emotional Connection subscales reflect the roles of social validation and affection in the promotion of well-being. The Influence subscale reflects the collective nature of well-being production in neighborhoods, where older adults are not merely recipients but potential contributors to community life (Wahl et al., 2012). This mapping of findings onto social production function theory strengthens the theoretical coherence of our results. Needs Fulfillment corresponds to goal-directed satisfaction of physical and social needs, Membership and Emotional Connection reflect behavioral confirmation and affection, and Influence represents opportunities for collective goal attainment. Together, these links suggest that sense of community supports multiple pathways to well-being.

Yet, while a strong sense of community offers a vital psychosocial resource, it is not in itself sufficient for the full realization of well-being in older age. As Nieboer and Cramm (2022) argue, this requires the development of solidarity, a shared commitment to mutual support and cooperation, within and between neighborhood groups. Solidarity enables the redistribution of resources and care, allowing older adults to thrive not only as individuals but as part of a community. These dynamics are especially important in heterogeneous urban neighborhoods where inequality and social fragmentation may limit opportunities for community participation (Phillipson, 2007). While the current study did not examine solidarity directly, future research should explore how solidarity norms and cross-cutting ties mediate the relationship between sense of community and well-being in later life.

## Limitations and future directions

Despite the strengths of our study in validating the BSCS among urban-dwelling older adults with diverse migration backgrounds, several limitations warrant discussion. First, our data were collected solely in Rotterdam, the Netherlands. This geographic focus may limit the generalizability of our findings to other urban contexts or rural areas, particularly in regions with different cultural, socioeconomic, or policy environments. Expanding data collection to include multiple cities or countries would help determine whether the observed psychometric properties and measurement invariance hold in varied settings (Vertovec, 2023). Follow-up studies should begin with careful piloting of the construct of sense of community itself to ensure that it is conceptually meaningful and culturally resonant in different contexts. While our validation suggests that the theoretical underpinnings of the instrument are robust and consistent across different ethnicities, it remains important to consider potential nuances in how community is understood and experienced in diverse sociocultural settings.

A second limitation is the exclusive focus on the four largest ethnic groups in the Netherlands: native-Dutch, Surinamese-Dutch, Turkish-Dutch, and Moroccan-Dutch. While these groups represent a significant portion of the Dutch population, including additional migrant and minority groups would enhance the external validity and cultural sensitivity of the BSCS, especially given that different communities may have distinct understandings and experiences of community influence and belonging.

We examined exclusion patterns across ethnic groups and found that differential exclusion due to limited language proficiency or extended stays abroad may have introduced some selection bias. Language difficulties appeared more often among Moroccan participants, while exclusions due to staying abroad or being in an institution were more common among Turkish and Moroccan participants compared to other groups. However, these exclusions involved relatively few individuals, making it unlikely that they had a substantial impact on the overall findings or their generalizability.

The Influence subscale showed somewhat lower internal consistency (*α* = 0.64), which is acceptable for a two-item scale and comparable to previous studies (Buckley et al., 2022; Yu et al., 2022). The modest reliability may reflect conceptual ambiguity, cultural differences in how neighborhood influence is perceived, or item wording that combines individual and collective aspects of agency. Cognitive interviewing could help determine how older adults interpret these items and whether refinements might strengthen the subscale since it would help clarify whether items are misunderstood, lack cultural resonance, or conflate distinct aspects of agency. Additionally, researchers might develop and test new items that explicitly differentiate between individual agency (e.g., “I can influence what happens in my building or street”) and collective efficacy (e.g., “People in my neighborhood can bring about change together”). These distinctions may help reduce variability driven by individual health, mobility, or experiences of exclusion.

Looking ahead, future studies would benefit from longitudinal designs that assess changes in the sense of community and its impact on health and well-being over time. Incorporating mixed-method approaches could provide deeper insight into how older adults develop, experience, and interpret their sense of community, particularly in relation to their shifting social roles and life circumstances. Moreover, given the growing diversification of urban populations, research that examines cross-cultural invariance and the mediating role of solidarity within communities could further enrich our understanding of how social ties and neighborhood environments contribute to successful aging (Nieboer & Cramm, 2022; Phillipson, 2007).

From a policy and practice perspective, the validated BSCS can serve as a valuable tool for local governments and community organizations aiming to foster age-friendly and inclusive neighborhoods. By reliably measuring sense of community across diverse ethnic groups, the scale can help identify areas where community and collective influence are weak, enabling targeted interventions to strengthen social ties and participation among older adults. This is especially important in multicultural urban settings, where policies must account for varied cultural understandings of community and inclusion. Moreover, regular use of the BSCS in community assessments can monitor the impact of programs designed to reduce social isolation and promote well-being. Practitioners should also consider the nuanced findings regarding the Influence subscale to develop strategies that empower older adults both as individuals and as part of collective neighborhood efforts, thereby enhancing their agency.

## Conclusion

The findings of this study confirm the BSCS as a reliable, valid, and culturally sensitive measure of the sense of community among community-dwelling older adults with native and migrant backgrounds. The measurement invariance analysis confirmed that the scale can be used effectively with diverse cultural groups, ensuring its broad applicability in various contexts. The findings contribute to a deeper understanding of well-being production in neighborhoods, particularly for older adults, by highlighting the effects of a sense of community on physical and social well-being in diverse environments. As such, the BSCS has the potential to advance research on the role of community in well-being achievement, offering valuable insights for local and national policy development. In an increasingly urbanizing and diversifying world, these insights can inform the development of inclusive, well-being-centered policies that address the needs of older adults of all backgrounds, fostering environments that support their independence, health, and social integration.

## Supplementary Material

gnaf239_Supplementary_Data

## Data Availability

The data used in this study are currently (2025) unavailable to other researchers, as this research is part of an ongoing project entitled “Community age-friendliness and well-being realization among older natives and people with Moroccan, Turkish, and Surinamese backgrounds in the Netherlands” (Nieboer & Cramm, 2022). Data will be shared 1 year after the completion of the project (2027) upon reasonable request. This study was not preregistered.
