# Supplementary material for: Psychometric properties of the brief sense of community scale for urban-dwelling older adults
Source: Gerontologist. 2025 Oct 27;65(12):gnaf239. doi: 10.1093/geront/gnaf239 (PMC12688443; doi:10.1093/geront/gnaf239)
Supplement: gnaf239_Supplementary_Data [file gnaf239_supplementary_data.zip › Bloem, Cramm, & Nieboer Suppl.docx]

## Appendix A: Brief Sense of Community Scale

| **Statement** |
| --- |
| 1. I can get what I need in this neighborhood. |
| 2. This neighborhood helps me fulfill my needs. |
| 3. I feel like a member of this neighborhood. |
| 4. I belong in this neighborhood. |
| 5. I have a say about what goes on in my neighborhood. |
| 6. People in this neighborhood are good at influencing each other. |
| 7. I feel connected to this neighborhood. |
| 8. I have a good bond with others in this neighborhood. |

## Appendix B: Dutch Translation of Brief Sense of Community Scale

| **Statement** |
| --- |
| 1. Wat ik nodig heb kan ik krijgen in deze buurt |
| 1. Deze buurt helpt me mijn behoeften te vervullen |
| 1. Ik voel me onderdeel van deze buurt |
| 1. Ik hoor bij deze buurt |
| 1. Ik heb inspraak over wat er in mijn buurt gebeurt |
| 1. Mensen in deze buurt beïnvloeden elkaar op een goede manier |
| 1. Ik voel me verbonden met deze buurt |
| 1. Ik heb een goede band met anderen in deze buurt |

## Appendix C: Turkish Translation of Brief Sense of Community Scale

| **Statement** |
| --- |
| 1. İhtiyacım olan şeyleri bu mahallede bulabiliyorum |
| 1. Bu mahalle ihtiyaçlarımı karşılamama yardımcı oluyor |
| 1. Kendimi bu mahallenin bir parçası hissediyorum |
| 1. Bu mahalleye kendimi ait hissediyorum |
| 1. Mahallemde olup bitenler hakkında söz hakkım var |
| 1. Bu mahalledeki insanların birbirleri üzerinde iyi etkisi bulunuyor |
| 1. Kendimi bu mahalleye bağlı hissediyorum |
| 1. Bu mahallede başkalarıyla iyi bir ilişkim var |

## Appendix D: Arabic Translation of Brief Sense of Community Scale

| **Statement** |
| --- |
| 1. يمكنني الحصول على كل ما احتاجه في هذا الحي |
| 1. يساعدني هذا الحي على تلبية احتياجاتي |
| 1. أشعر أنني جزء من هذا الحي |
| 1. أشعر أنني أنتمي إلى هذا الحي |
| 1. يمكنني إبداء رأيي فيما يحدث في الحي |
| 1. تؤثر الناس في هذا الحي على بعضهم البعض بطريقة جيدة |
| 1. أشعر بالارتباط بهذا الحي |
| 1. العلاقة بينني وبين الآخرين في هذا الحي جيدة |
